# External auditory exostoses in the Xuchang and Xujiayao human remains: Patterns and implications among eastern Eurasian Middle and Late Pleistocene crania

**DOI:** 10.1371/journal.pone.0189390

**Published:** 2017-12-12

**Authors:** Erik Trinkaus, Xiu-Jie Wu

**Affiliations:** 1 Department of Anthropology, Washington University, Saint Louis, MO, United States of America; 2 Key Laboratory of Vertebrate Evolution and Human Origins, Institute of Vertebrate Paleontology and Paleoanthropology, Chinese Academy of Sciences, Beijing, China; University of Delaware, UNITED STATES

## Abstract

In the context of Middle and Late Pleistocene eastern Eurasian human crania, the external auditory exostoses (EAE) of the late archaic Xuchang 1 and 2 and the Xujiayao 15 early Late Pleistocene human temporal bones are described. Xujiayao 15 has small EAE (Grade 1), Xuchang 1 presents bilateral medium EAE (Grade 2), and Xuchang 2 exhibits bilaterally large EAE (Grade 3), especially on the right side. These cranial remains join the other eastern Eurasian later Pleistocene humans in providing frequencies of 61% (N = 18) and 58% (N = 12) respectively for archaic and early modern human samples. These values are near the upper limits of recent human frequencies, and they imply frequent aquatic exposure among these Pleistocene humans. In addition, the medial extents of the Xuchang 1 and 2 EAE would have impinged on their tympanic membranes, and the large EAE of Xuchang 2 would have resulted in cerumen impaction. Both effects would have produced conductive hearing loss, a serious impairment in a Pleistocene foraging context.

## Introduction

Dense bony growths protruding into the external auditory canal, external auditory exostoses (EAE), have been documented clinically for more than a century (e.g., [1,2]), known anthropologically since at least the analyses of Hrdlička [3], and summarized extensively more recently [4–6]. They are usually rounded hyperostotic protrusions into the meatus that may partially or largely occlude the canal. Clinical and bioarcheological observations indicate that they are principally due to an environmental irritation of the mucoperiosteum of the canal (see [4,6–8]). The most frequently observed irritant is cold water, in the context of cold water sports and foraging. However, they may occur in a variety of contexts, given sufficient inflammation of the soft tissue lining of the auditory canal. They are often benign but can lead to cerumen impaction and conductive hearing loss.

EAE were first observed in human fossil remains by Boule [9] in the La Chapelle-aux-Saints 1 Neandertal, and subsequently by Weidenreich [10] in the Middle Pleistocene Zhoukoudian Skull 10. More recently, EAE presence has been noted in several western Eurasian Middle and Late Pleistocene archaic humans [11–14] and in a few Late Pleistocene early modern humans [15], and their absence noted in several more [16–18]. Moreover, EAE were recently noted in the two early Late Pleistocene partial neurocrania from Xuchang [19], but they have not been mentioned for the similarly aged Xujiayao 15 temporal bone [20]. Given these paleopathological and distributional considerations, we present here these bony auditory growths in the Xuchang and Xujiayao late archaic humans, and evaluate their implications in the contexts of the available Middle and Late Pleistocene eastern Eurasian human remains and the patterns of frequencies across recent human skeletal samples.

## Materials and methods

### Xuchang 1 and 2

The Xuchang 1 and 2 crania were excavated *in situ* in Layer 11 of the Lingjing site in Xuchang county, Henan Province, northern China (34° 04’ 08.6” N, 113° 40’ 47.5” E) between 2007 and 2014 [19]. Layer 11 has yielded a diverse late Middle to early Late Pleistocene macromammalian fauna [21,22], a Middle Paleolithic lithic and bone assemblage [23,24], and a consistent series of optically stimulated luminescence (OSL) dates placing Layer 11 between 125,000 and 105,000 years ago [19]. The overlying Layers 10 and 9 have provided OSL determinations between 100,000 and 90,000 years ago, substantiating the early MIS 5 (MIS 5e or 5d) age of the human crania.

Xuchang 1 and 2 consist of partial neurocrania, that of the former being more complete [19]. Each cranium retains the posterior cranial base from right to left temporal bones. Xuchang 1 has most of the vault from the supraorbital torus posteriorly, but Xuchang 2 preserves primarily occipital and posterior parietal bones in addition to the temporal bones. The crania were excavated as 26 and 16 pieces respectively, and the elements have been kept separate, the crania having been reconstructed virtually and with casts. They derive from young adults (based on relatively open cranial vault sutures), and there is no evidence of abnormalities on them, other than the EAE presented here. They are considered as eastern Eurasian late archaic humans given their low and broad neurocrania with the maximum breadth inferiorly, the presence of supraorbital and nuchal tori, a “Neandertal” suprainiac fossa, and their labyrinthine proportions [19].

**Fig 1:**
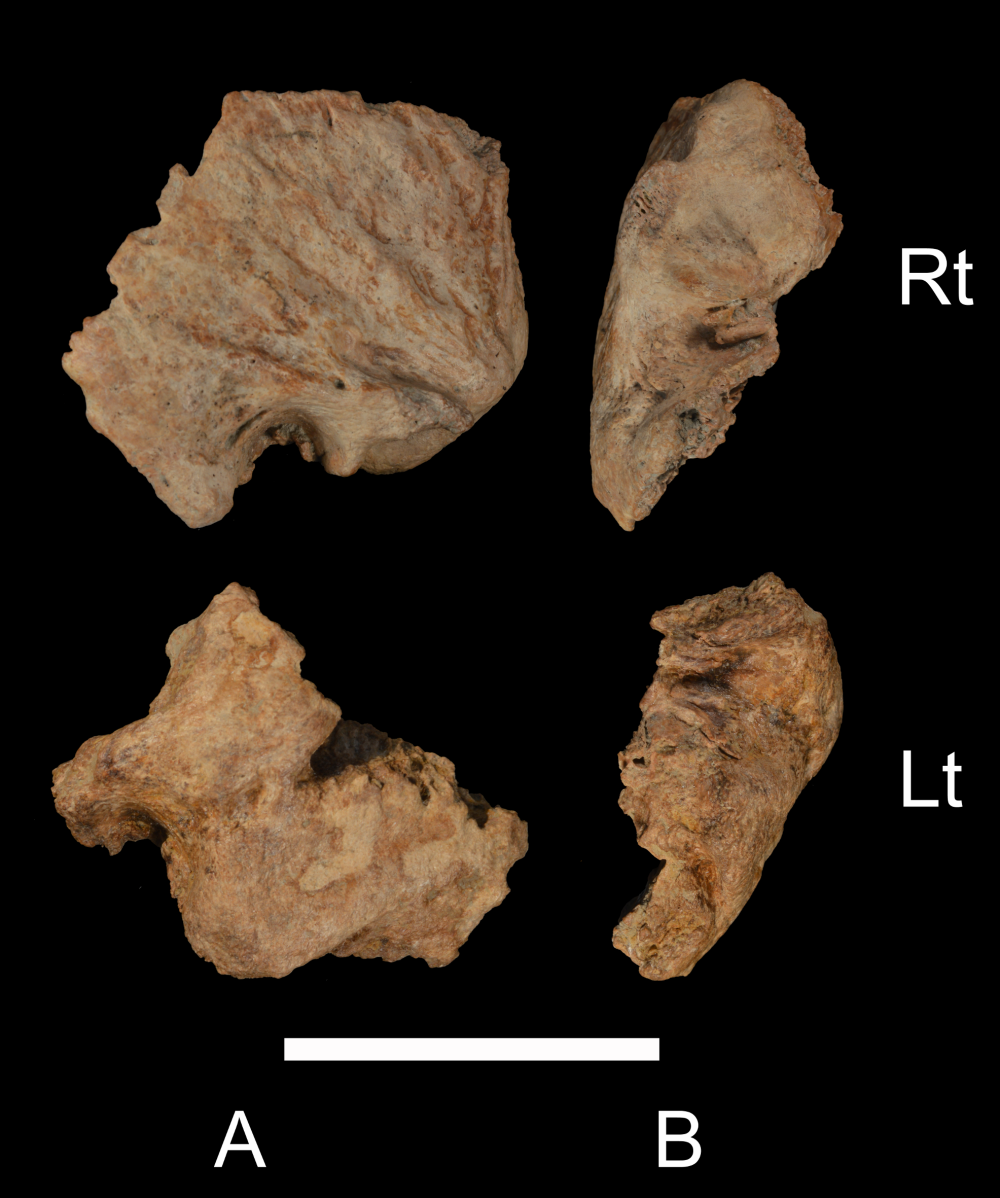
Xuchang 1 temporal bones. A: lateral views of the preserved portions. B: inferior views of the preserved portions. Rt: right; Lt: left. Scale bar: 5 cm.

The Xuchang 1 right temporal bone (#7019; Fig 1) has the auditory canal from the (lateral) porus to the attachment area of the tympanic membrane (mediolateral breadth of the canal: 22 mm), but it consists only of the roof from the complete postglenoid process to the anterior mastoid process. Only the posterosuperior edge of the tympanic bone remains, adjacent to the mastoid process. The left temporal bone (#7016; Fig 1) preserves the auditory canal only along its roof from the postglenoid process to the mastoid process, with a posterolateral portion of the tympanic bone adherent to the anterior mastoid process. It is also present from the tympanic membrane attachment area to the lateral extent of the canal (mediolateral breadth of the canal: 21 mm).

**Fig 2:**
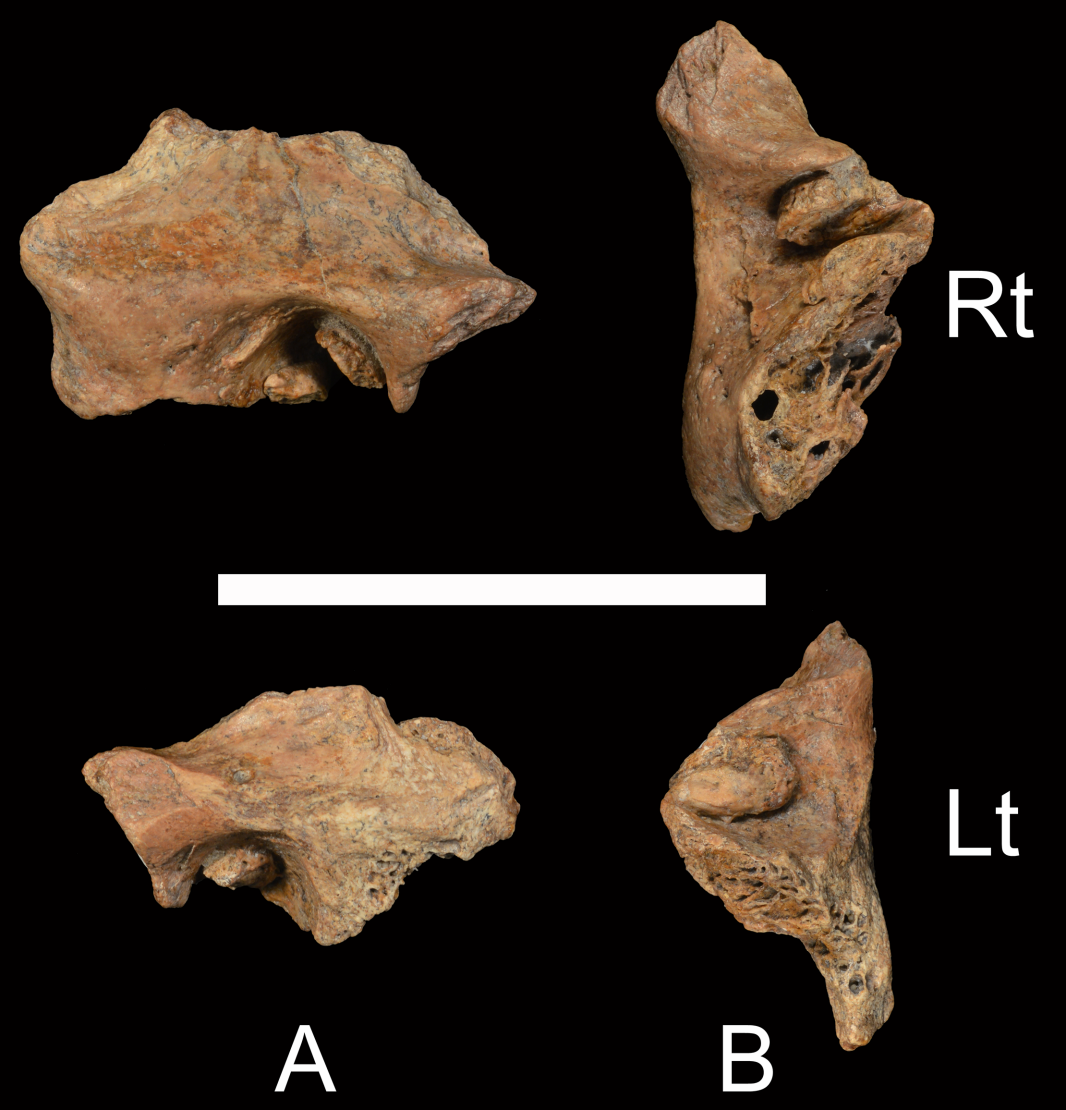
Xuchang 2 temporal bones. A: lateral views of the preserved portions. B: inferior views of the preserved portions. Rt: right; Lt: left. Scale bar: 5 cm.

The Xuchang 2 right and left temporal bones (#14004 and #14005 respectively; Fig 2) preserve their auditory canals to a similar extent as those of Xuchang 1, in that each extends from the complete postglenoid process to the anterior mastoid process and from the tympanic membrane attachment area medially to the lateral porus. They lack the tympanic bones entirely. The auditory canals are 19.5 to 20 mm mediolateral.

### Xujiayao 15

The Xujiayao 15 temporal bone (#PA1498) was discovered during excavations in 1979 at Locality 74093 (40° 06′ 02″ N, 113° 58′ 39″ E) of the Xujiayao site complex in Houjiayao village, northwestern Nihewan Basin, northern China. The Xujiayao locality is an open-air site comprised of fluviolacustrine deposits. The paleoanthropological remains derive from sediments that have been dated to the early Late Pleistocene (most likely Marine Isotope Stage 5) based on associated faunal species [25,26], six Uranium-series dates on *Equus* sp. and *Coelodonta antiquitatis* tooth enamel (≈104 to ≈125 ka BP) [27], and the presence of an underlying paleomagnetic reversal that most likely represents the Blake Excursion ≈123 ka BP [28,29]. Suggestions of an earlier (Middle Pleistocene) age from the sedimentary paleomagnetism [30] are based on unverifiable assumptions of sedimentation rates, and there is uncertain stratigraphic correlation between a recently excavated late Middle Pleistocene section and the deposits that yielded the human remains [31]. The Xujiayao human fossils consist of 17 pieces of immature and mature craniofacial remains and isolated teeth, which probably represent a smaller number of individuals.

**Fig 3:**
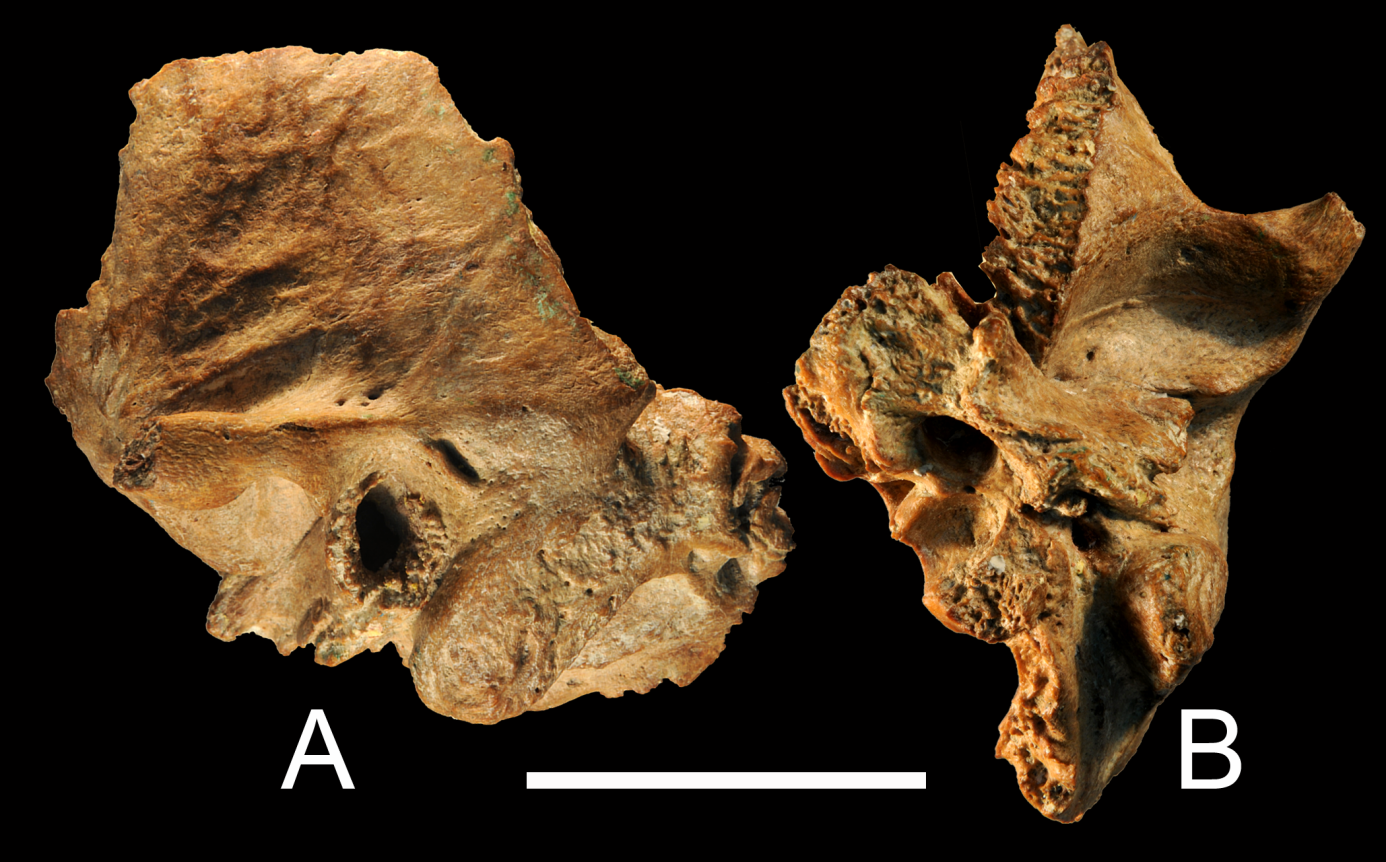
Xujiayao 15 left temporal bone. A: lateral; B: inferior. Only the zygomatic process, the medial petrous tip, and small chips along the squamous suture are absent; a sutural bone was likely present anterosuperiorly. Scale bar: 5 cm.

Xujiayao 15 is a largely complete and isolated left temporal bone ([20,32]; Fig 3). The portions making up the external auditory canal are complete and undamaged. The foramen of Huschke is closed, but there is a fusion line along the inferior tympanic bone, open medially and laterally. The Xujiayao sample is aligned with other eastern Eurasian late archaic humans in terms of its labyrinthine proportions, nuchal tori, nasal floor and mandibular ramal morphology, and dental morphological details [32–36].

### Comparative samples

**Fig 4:**
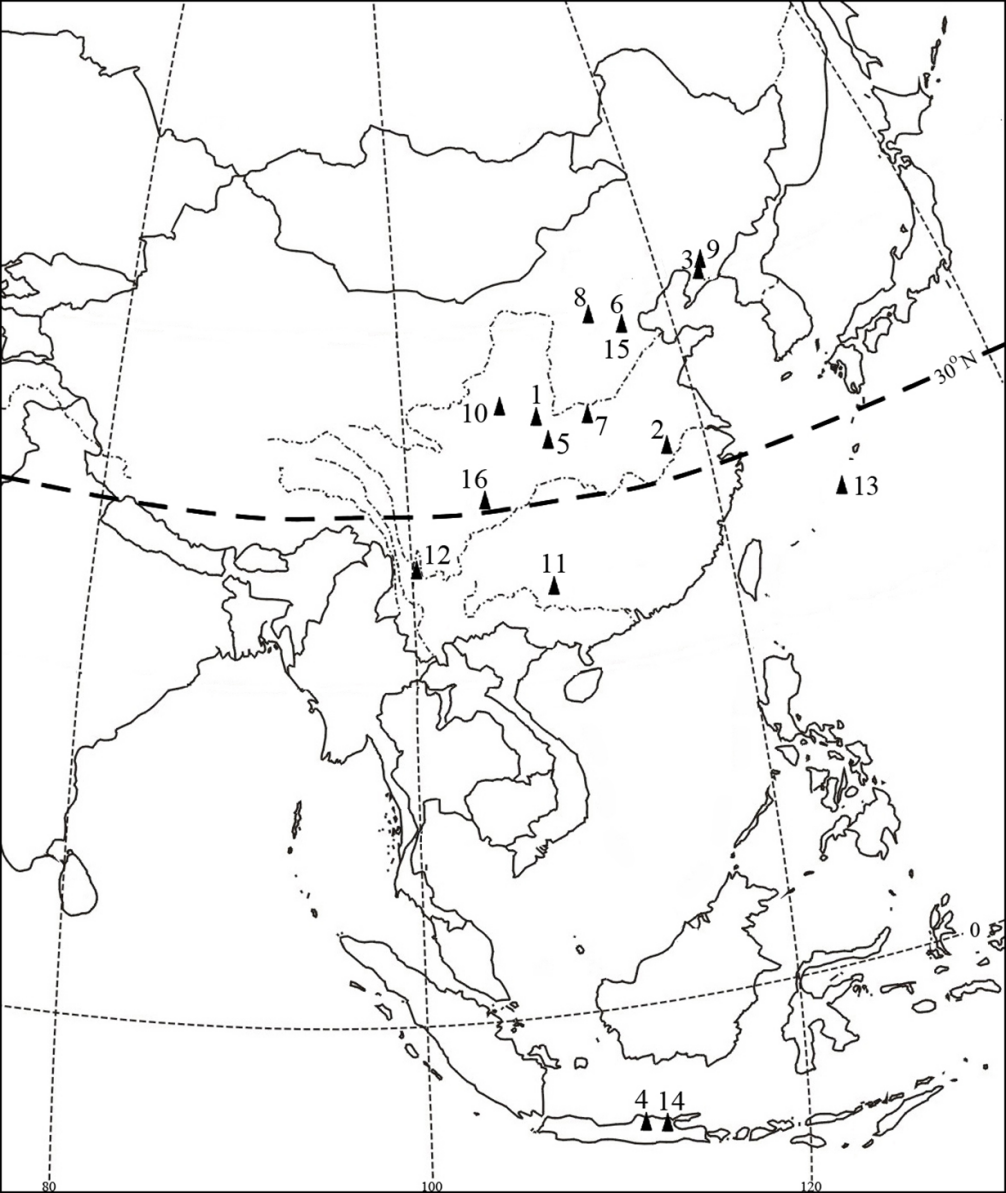
Eastern Eurasian middle and late Pleistocene sites yielding crania preserving at least one auditory meatus sufficiently intact to provide information on external auditory exostoses. 1: Dali; 2: Hexian; 3: Jinniushan; 4: Ngandong; 5: Yunxian; 6: Zhoukoudian Loc. 1; 7: Xuchang; 8: Xujiayao; 9: Gezi Cave; 10: Jingcuan; 11: Liujiang; 12: Lijiang; 13: Minatogawa; 14: Wajak; 15: ZKD-Upper Cave; 16: Ziyang.

To provide a context for the Xujiayao and Xuchang EAE, data have been collected for two samples, a regional paleontological one (S1 Text) and a global recent human one (S2 Text). The fossil sample (Table 1; Fig 4) includes the available and sufficiently preserved Middle and Late Pleistocene temporal bones from eastern Eurasia, including China, Okinawa (Japan) and Indonesia. The archaic fossils derive from the Middle Pleistocene sites of Dali, Hexian, Jinniushan, Ngandong, Yunxian, and Zhoukoudian Locality 1 (ZKD) (S1 Text). The early modern human EAE data are from the Late Pleistocene Gezi, Jingcuan, Lijiang, Liujiang, Minatogawa, Wajak, and Zhoukoudian-Upper Cave (ZKD-UC) crania (S1 Text). The Xuchang, Xuchiayao, Hexian, Jingcuan, Yunxian, Gezi and Lijiang observations are from visual inspection of their temporal bones. The Dali and Liujiang ones are from CT scans of the crania. The ZKD observations are from Weidenreich’s [10] descriptions, combined with his photographs and casts, and the Ngandong observations are similarly from Weidenreich [37], Santa Luca [38] and casts. The Minatogawa ones are from Suzuki’s [15] descriptions, combined with his photographs and a cast of Minatogawa 1. The Wajak 1 score is from a photograph of the temporal bone, and the Jinniushan and ZKD-UC observations are based on casts.

**Table 1:** The eastern Eurasian Pleistocene remains providing external auditory exostosis observations and their contexts.

*Site*	*Location*	*Site form*	*Latitude*
**Middle Pleistocene**			
Dali	Shaanxi, China	river terrace gulley	35° 52’ N
Hexian	Anhui, China	cave	31° 45’ N
Jinniushan	Liaongning, China	cave	40° 34’ N
Ngandong	Java, Indonesia	fluviatile terrace	7° 18’ S
Yunxian	Hubei, China	river terrace	31° 51’ N
Zhoukoudian Loc. 1	Beijing, China	cave	39° 41’ N
**Early Late Pleistocene**			
Xuchang	Henan, China	lacustrine	34° 04’ N
Xujiayao	Hebei-Shanxi, China	fluviatile/lacustrine	40° 06’ N
**Upper Paleolithic**			
Gezi Cave	Liaoning, China	cave	41° 15’ N
Jingcuan	Gansu, China	gulley near river	35° 23’ N
Lijiang	Yunnan, China	fluviatile	26° 47’ N
Liujiang	Guangxi, China	cave	24° 10’ N
Minatogawa	Okinawa, Japan	fissure fill	26° 07’ N
Wajak	Java, Indonesia	cave	8° 06’ S
ZKD-Upper Cave	Beijing, China	cave	39° 41’ N
Ziyang	Sicuan, China	fluviatile	30° 07’ N

The recent human global sample consists primarily of EAE presence/absence frequencies from 114 samples. The majority of the data (57%) are from Kennedy [4], to which have been added data from additional and more recent sources (S2 Text). Only samples with N ≥ 30 have been included, and in several cases closely related smaller samples (i.e., from neighboring archeological sites of a given period/culture) have been pooled. Male and female data when available are combined, given the absence of reliable sex assessments for all but one (Jinniushan 1) of the archaic human remains and many of the early modern humans. Moveover, sex-specific frequencies are not available for a substantial number of the recent human comparative samples [4]; it is recognized that male frequencies are often higher than those of the females, but this should have little effect if the male-female proportions are generally similar across the samples and/or the sample frequencies are low. Only late adolescent and adult data are included; although the frequency or severity of EAE, as a degnerative process, should increase with age, they can occur at any age after mid-adolescence. Moreover, reliable adult ages-at-death are not available for most of the fossil specimens or recent human samples.

Following Kennedy [4], the samples are allocated to low (<30°), middle (30°–45°), and high (>45°) latitude samples. In addition, because there is abundant evidence for the proximity to water and aquatic resource exploitation to be involved in the etiology of EAE ([6]; see below), the latitudinal samples are divided into “dry” (terrestrial) and “wet” (maritime and riverine) samples (considering evidence of aquatic resource exploitation when available, and bearing in mind that the “wet”/“dry” distinction is not always evident) (see S2 Text).

More limited recent human comparative data exist for the frequencies of grades of EAE. The available data are provided in S2 Text. Although the paleontological samples span the low and middle latitudes (Table 1), they are not so separated in terms of frequencies (Table 2) given their modest numbers (S1 Text).

**Table 2:** Distribution of external auditory exostosis grades among middle and late Pleistocene eastern Eurasian archaic [without and with Xuchang (XUC) and Xujiayao (XJY)] and early modern humans. Paleopathological details are in the text and S1 Text.

	Grade 0	Grade 1	Grade 2	Grade 3	N
Eastern archaic humans	46.7%	46.7%	6.7%	—	15
Eastern archaics plus XUC & XJY	38.9%	44.4%	11.1%	5.6%	18
Eastern early modern humans	41.7%	50.0%	8.3%	—	12

### Methods

External auditory exostoses are identified as growths into the auditory canal from its sqamous and/or tympanic portions [5,8,39]. They are usually rounded protrusions and may occur as single or multiple growths. They normally do not develop from the tympanosquamous or tympanomastoid sutures; such sutural protrusions are osteomata, or benign neoplasms, which normally occur laterally within the meatus, are less frequent, and are often solitary [8,39]. It is possible that osteomata have been included within some of the comparative data [5], but every effort has been made to distinguish between exostoses and osteomata, and to exclude the latter.

The EAE were scored using the four part ordinal scale of Cooper et al. [40], Crowe et al. [41] and Villotte et al. [42] (see also [5]). Grade 0 indicates the absence of EAE, Grade 1 indicates small mildly protruding single or multiple exostoses, and Grade 2 reflects one or more large EAE projecting well into the auditory canal. Grade 3 indicates a meatus which is largely or completely blocked by exostosis growth. A number of the Pleistocene tympanic bones are thickened at their lateral margins, especially where the inferior tympanic crest meets the porus. This thickening is not considered to be part of the EAE, which consist of bony growths into the meatus.

The grades for the comparative eastern Eurasian paleontological specimens are in S1 Text, along with brief descriptions of the growth(s), and summary frequencies for the grades of the paleontological and available recent human samples are in Table 2 and S2 Text. For those fossil specimens that adequately preserve both temporal bones, grades for both sides of specimen are provided in S1 Text. For the cases in which a cranium is asymmetrical in its grade, the grade of the side with the larger exostoses is employed for that specimen in the computation of sample frequencies by grade (Table 2) to provide an indication of maximum response. There is some degree of interobserver error in these qualitative ordinal grades [6], but it is generally small (<2% in one analysis [41]).

The Xujiayao 15 retains its entire auditory canal, and hence complete observations are possible for it. The Xuchang meatus, however, all lack the tympanic bone, such that observations are limited to the superior halves of the canals. These descriptions of the Xuchang canals therefore represent a conservative assessment of their EAE development.

## Results

### Xuchang 1

**Fig 5:**
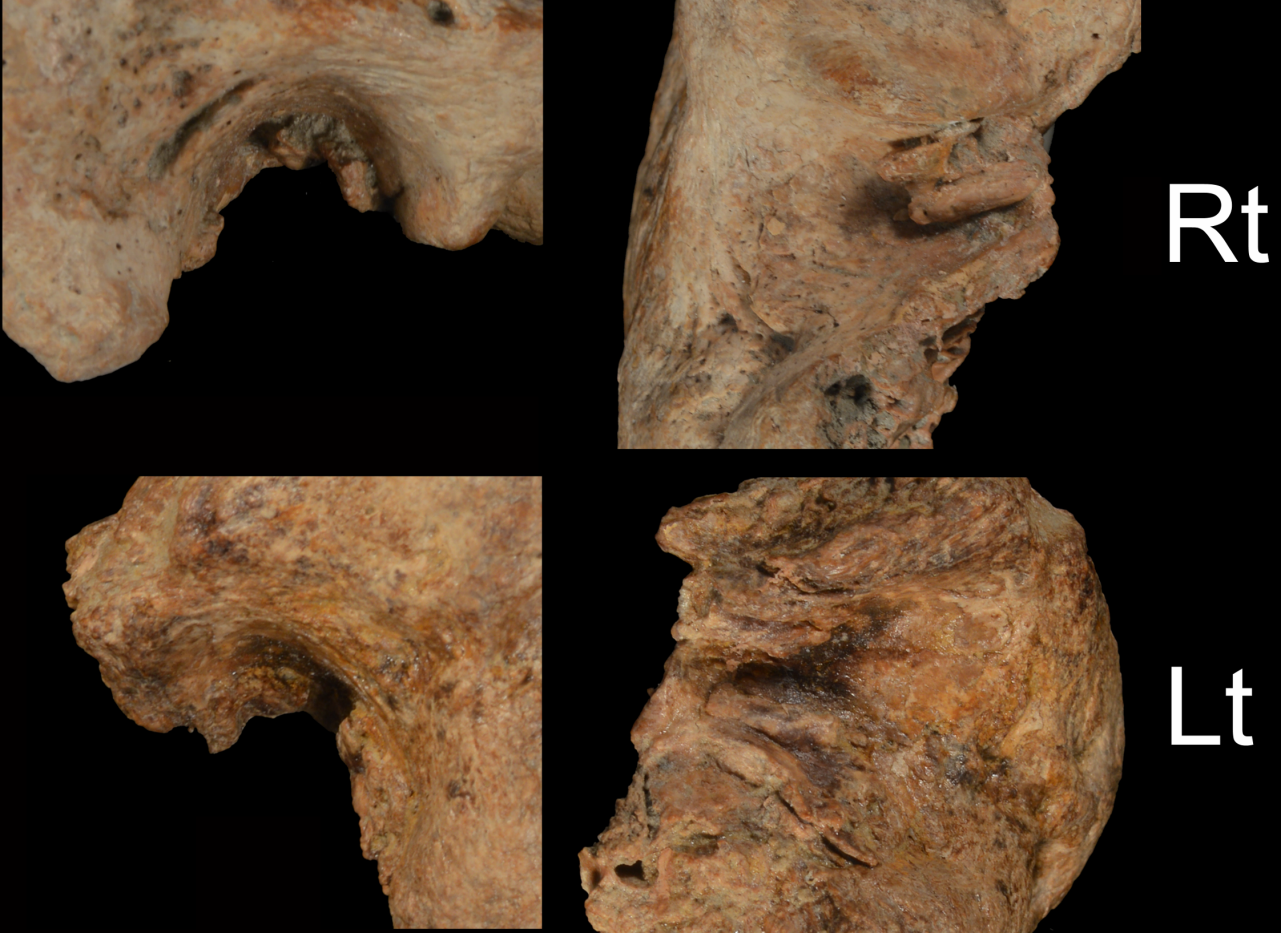
Xuchang 1 auditory meatus. Left: lateral views of the external auditory canals. Right: inferior views of the superior auditory canals. Rt: right; Lt: left. Note that only the superior portions of the canals are preserved, but they extend from the lateral margins to the original positions of the tympanic membranes. Not to scale.

The Xuchang 1 right auditory meatus (Fig 5) has a 9 mm long rod-like growth, 2 mm in diameter, running mediolaterally along the superior apex of the canal, from the tympanic membrane to near the mediolateral middle of the canal. At the medial end of the canal, there is a small rounded knob of bone between the rod and the posterior edge of the canal. Anterior to the rod along the apex of the canal, there is a more irregular growth of new bone, 6.7 mm mediolateral and 6.0 mm superoinferior. It extends close to the inferior end of the postglenoid process. In addition, posteriorly close to where the tympanic bone would have articulated with the anterior mastoid process, there is an exostosis (most evident in lateral view) projecting 1–2 mm into the canal. This posterior growth tapers off as it extends towards the medial end of the canal.

The left meatus of Xuchang 1 (Fig 5) has a rod-like growth, 2 mm wide and 8.3 mm long, on the middle of the postglenoid process and extending laterally from the area of the tympanic membrane. There is also a curved, rod-like growth, 8 mm mediolateral, in the anterosuperior canal, also extending laterally from the medial canal. The middle of the roof of the canal is smooth, but the posterior wall has a broader growth, 3.5 mm wide at its medial end and expanding to 8.2 mm wide laterally, and extending 15 mm from the tympanic membrane area. This posterior broader growth appears as a series of small rods of bones along the medial 6 mm, which then blend into a wider growth laterally.

When both Xuchang 1 meatus are viewed laterally (Fig 5), they appear to impinge moderately on the auditory canal, but collectively they substantially reduce the size of the canal. Any further exostoses from the absent tympanic bones would only increase the constriction. They should therefore be scored as Grade 2. In addition, the medial ends of the exostoses on both sides substantially reduced the area of the tympanic membrane, and they are likely to have impinged on the membranes.

### Xuchang 2

**Fig 6:**
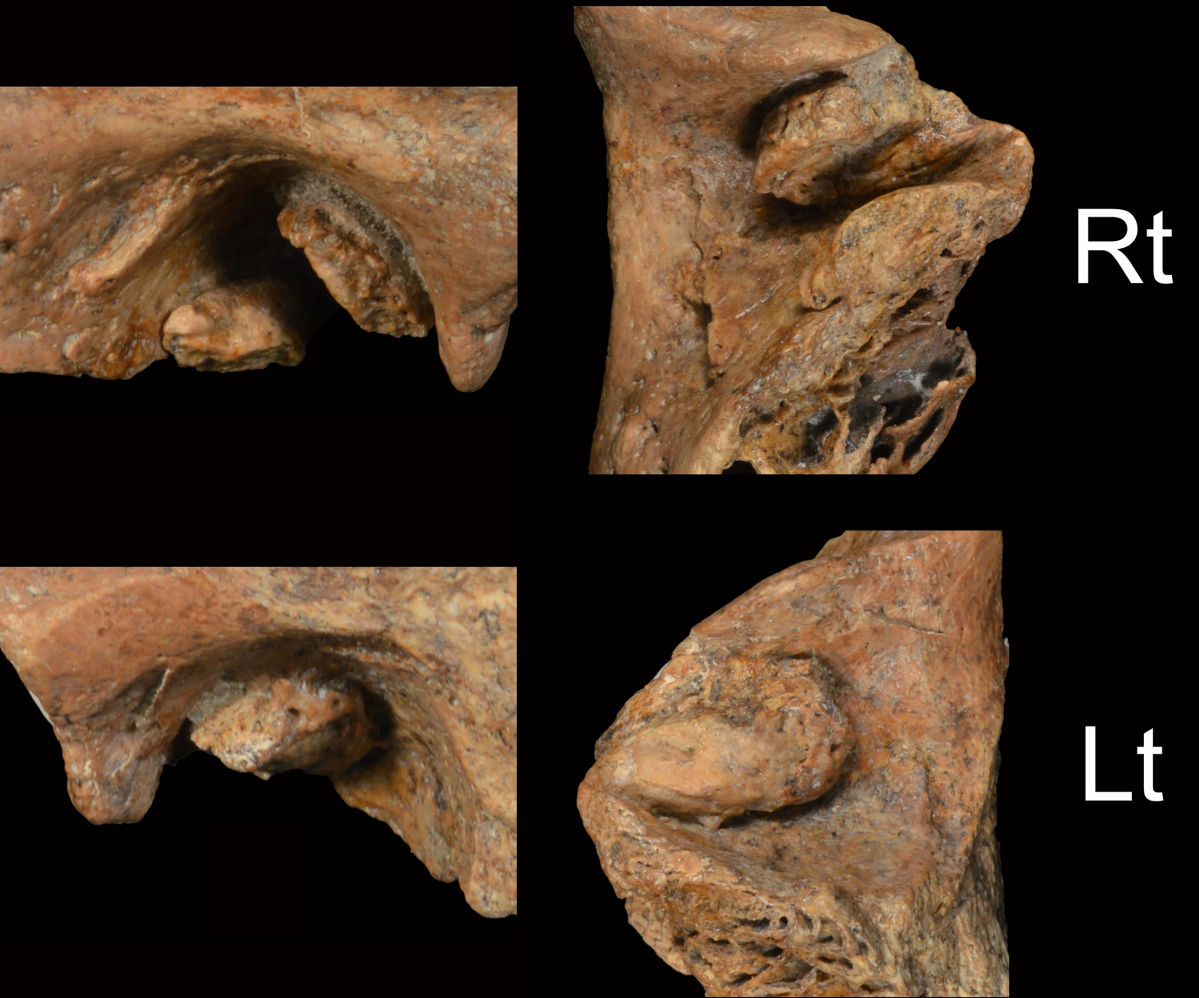
Xuchang 2 auditory meatus. Left: lateral views of the external auditory canals. Right: inferior views of the superior auditory canals. Rt: right; Lt: left. Note that only the superior portions of the canals are preserved, but they extend from the lateral margins to the original positions of the tympanic membranes. Not to scale.

The right auditory canal of Xuchang 2 (Fig 6) presents two substantial exostoses. The larger one is along the anterosuperior meatus, extending from just above the inferior apex of the postglenoid process to the superior apex of the canal roof. It is evenly ≈3 mm thick, has a maximum breadth (posterosuperior-to-anteroinferior) of 8.5 mm, and extends 14.5 mm (74% of the canal length) from the tympanic membrane attachment. The second growth is along the posteroinferior break of the bone, and it lacks its inferior extent due to damage to its inferior edge. It also extends 14.5 mm from the medial canal and is ≈3 mm in thickness. At the medial end of the auditory canal, these two exostoses reduce the superior diameter of the canal from ≈8 mm to ≈3 mm in the middle of the canal and to 2.3 mm at the roof of the canal (a diameter reduction to ≈38%; or a reduction to ≈14% of the cross-sectional area if the observable portion of the auditory meatus is representative of the canal as a whole).

The left canal of Xuchang 2 only has a single exostosis attached to the roof of the canal (Fig 6). It is rod-like on its medial half and more irregular and porous laterally. It extends 13 mm from the tympanic membrane area, has a maximum anteroposterior breadth in the middle of 7.5 mm, and has a maximum superoinferior projection of ≈3.5 mm at its lateral end. There is no evidence of further growth on the preserved anterior or posterior portions.

The two large exostoses of the right canal, even without any further exostoses on the missing tympanic bone, qualify it as Grade 3. The single large one on the superior left canal, if the only one that was present, would provide at least a Grade 2; any further tympanic growths, however, would qualify it as Grade 3. Moreover, as with the growths in the Xuchang 1 meatus, those of Xuchang 2 would have impinged on the tympanic membrane, given their medial extents, especially the more anterior growth in the right canal.

### Xujiayao 15

**Fig 7:**
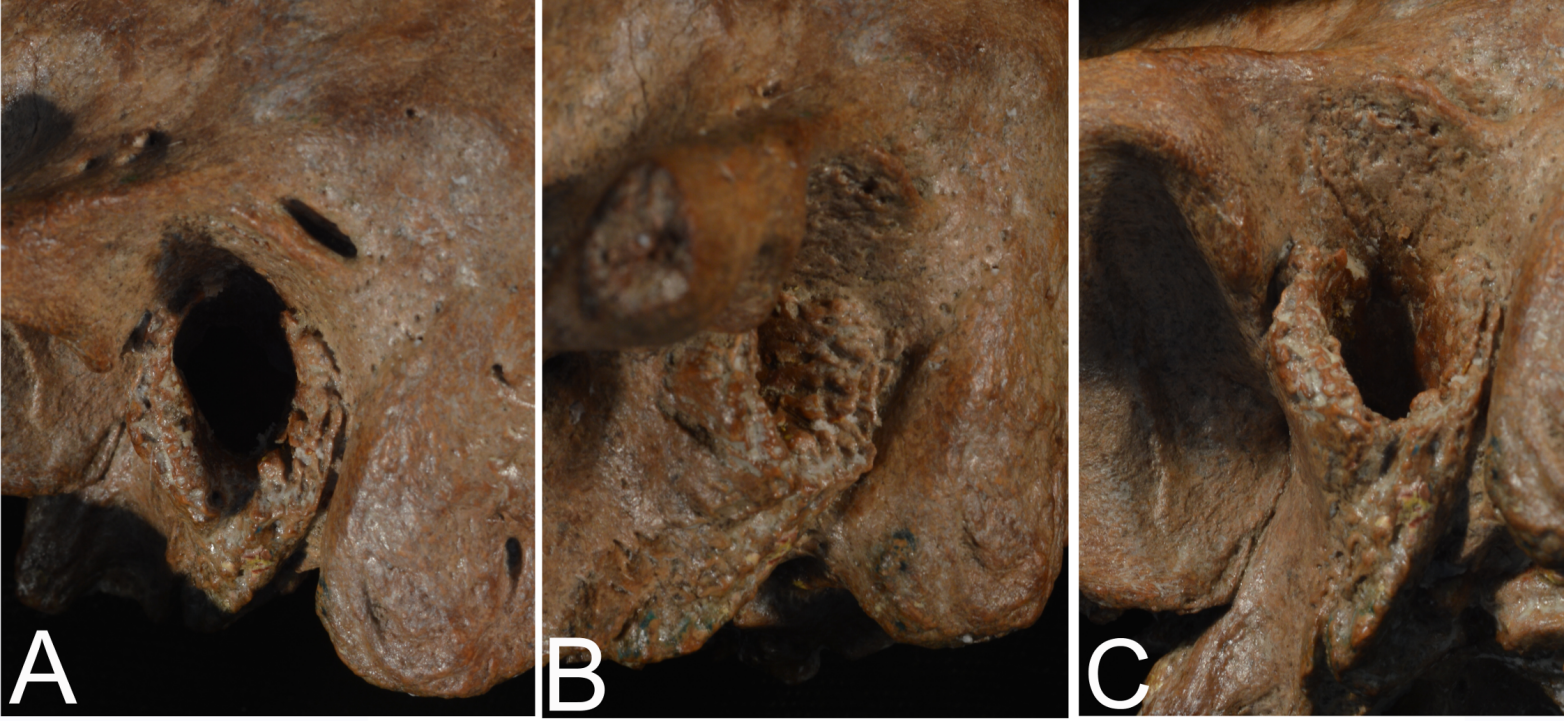
Xujiayao 15 left auditory meatus and porus. A: lateral view of the auditory porus and anterior mastoid process. B: anterior view of the posterior portion of the lateral tympanic bone, showing the irregularities of the surface. C: inferior view of the superior (squamous) portion of the auditory porus, with the porous new bone extending across the superior portion and extending into the meatus. Scale bar: 5 cm.

In contrast, the Xujiayao 15 auditory canal has little impingement of lumen (Fig 7A). The lateral tympanic bone is anterosuperiorly narrow (1.8 mm), then thickens through the anteroinferior potion (4.1 mm), and then thins slightly inferiorly (3.5 mm). Its meatal surface is smooth anteriorly, but it becomes more irregular inferiorly and especially posteroinferiorly (Fig 7B), the changes extending 13–15 mm into the canal. On both the lateral margin and into the meatus there is a series of small knobs of bone. The variable thickening of the tympanic bone is normal variation, especially in Pleistocene archaic humans [10], but the irregular surface and series of small knobs along the posterior and posteroinferior tympanic bone into the canal represents a minor degree of extra growths into the canal. These minor growths indicate a Grade 1 EAE formation for Xujiayao 15.

In addition, there is an area of new porous bone along the superior opening (Fig 7C). lateral of the meatus but covering the superior portion of the porus. It is not properly an EAE, but it indicates additional inflammation of the auditory canal region.

### Comparative assessments

**Fig 8:**
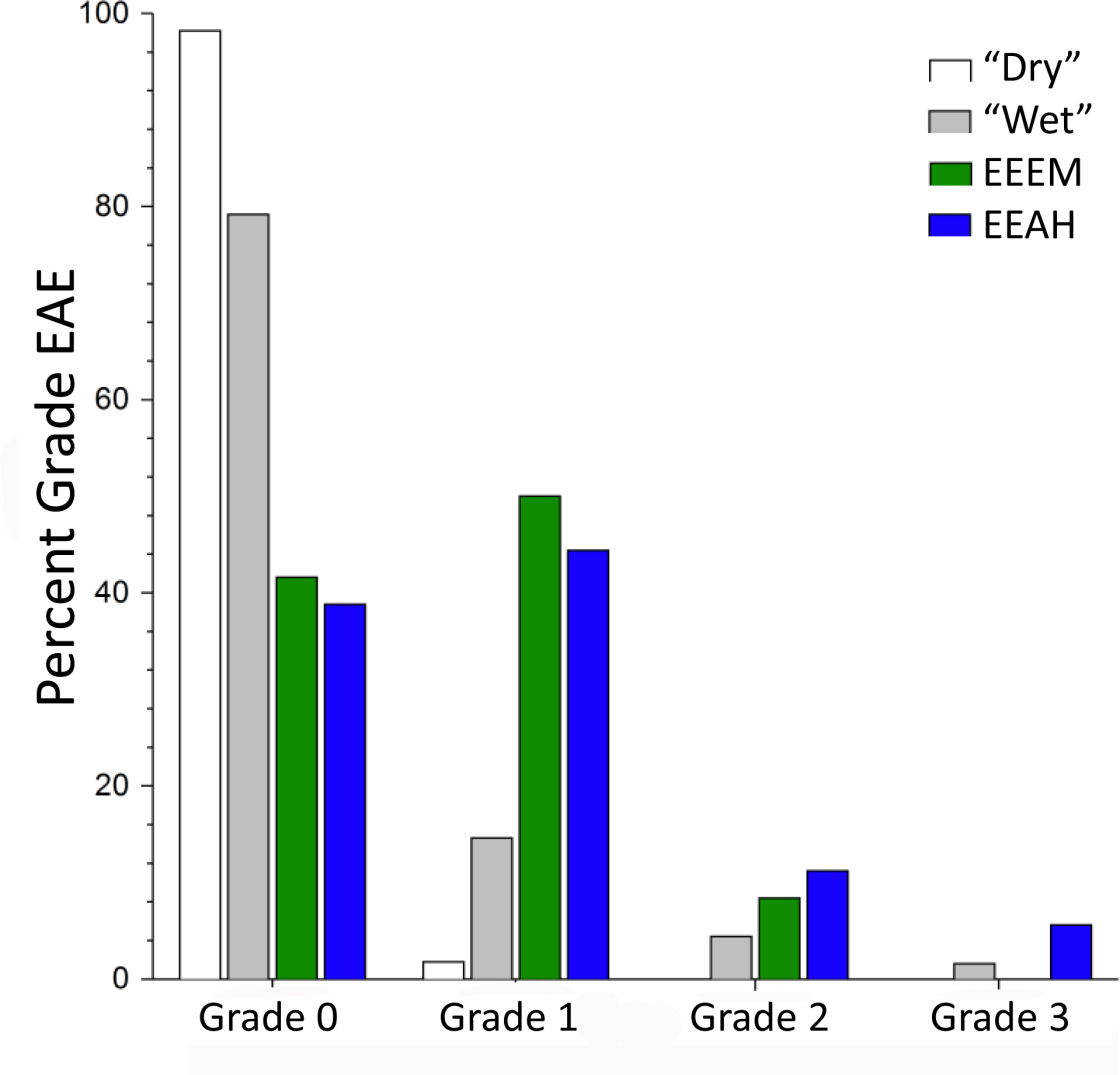
Distributions of the 4 grades of external auditory exostosis formation. The bars are for the recent human “dry” and “wet” samples (S2 Text) and for the eastern Eurasian early modern (EEEM) and archaic (EEAH) human samples (see Table 2). The EEAH sample includes the Xuchang and Xujiayao specimens.

The Xuchang 1 and Xujiayao 15 EAE are unexceptional in the context of Middle and Late Pleistocene eastern Eurasian humans. Moderately large ones (Grade 2) are present in ZKD Skull 10 and in Minatogawa 2, and 46.7% and 50.0% respectively of the archaic and early modern samples exhibit small (Grade 1) exostoses (Table 2, S1 Text). Grade 2 exostoses are also present in four western Eurasian archaic humans, La Chapelle-aux-Saints 1, Krapina 39.1, Spy 1 and Tabun 1 [14]. The very large (Grade 3) ones of Xuchang 2 are relatively unusual. None of the other eastern Eurasian Pleistocene crania have ones as large, and only one other Pleistocene human (the Shanidar 1 Neandertal) has EAE that are larger [14]. Among recent human samples for which grade frequencies are available (Fig 8; S2 Text), less than half of the samples exhibit any Grade 3 EAE, and the frequencies are ≈5% or less.

**Fig 9:**
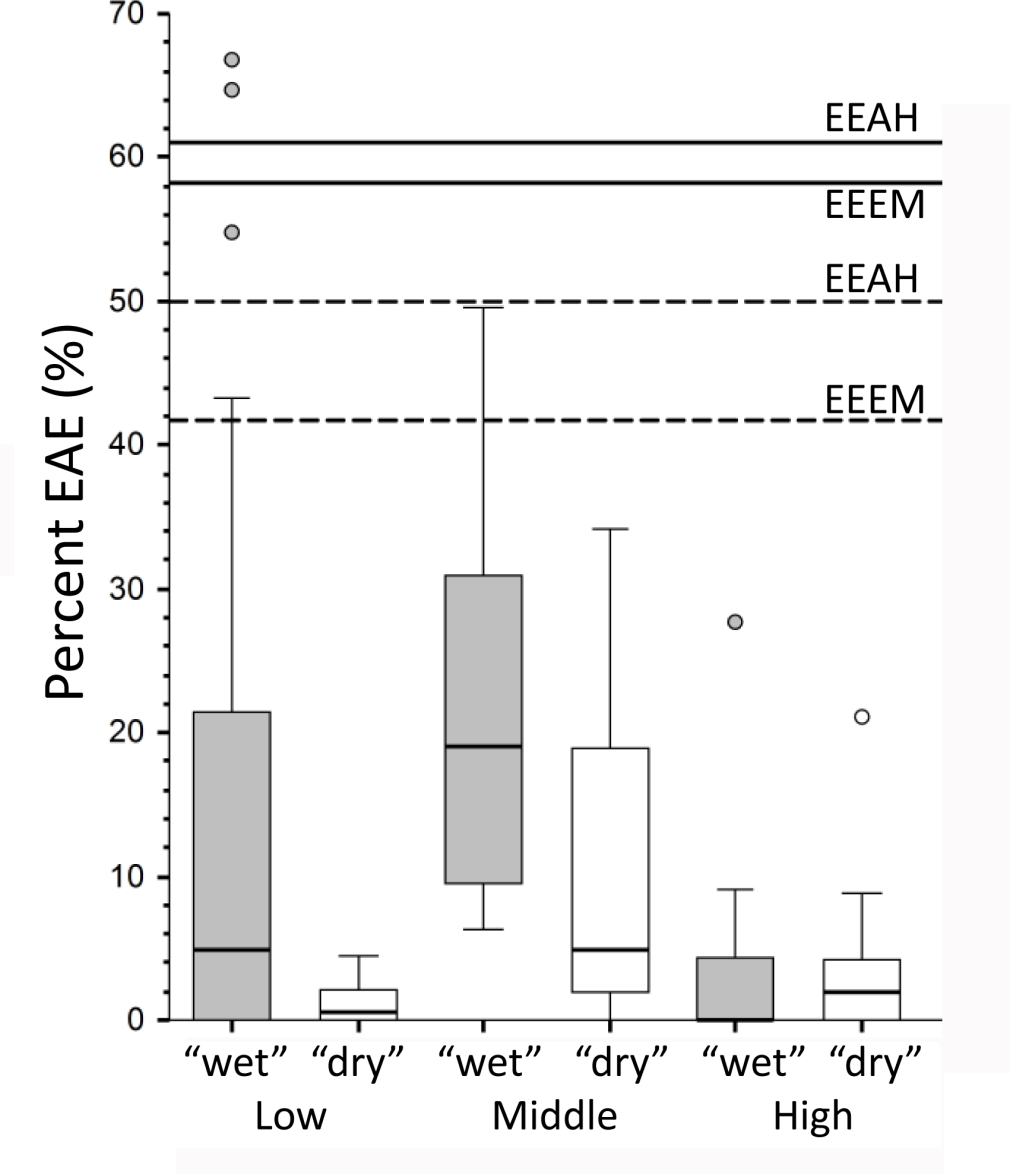
Frequencies of external auditory exostosis (EAE) presence. Box plots for the global recent human samples, with the frequencies for the eastern Eurasian archaic and early modern human samples. The recent human samples are separated into Low (<30°), Middle (30° - 45°) and High (>45°) latitude samples, each separated into coastal/riverine (“wet”) versus inland/terrestrial (“dry”) samples. The numbers of samples, from low and “wet” to high and “dry” are: 30, 22, 14, 19, 13 and 16. The lines are for the maximum eastern Eurasian archaic (EEAH) and early modern (EEEM) human samples (solid lines) and the more conservative ones removing specimens with very small EAE from the frequencies (dashed lines) (see text).

At the same time, the overall frequencies of the eastern Eurasian Pleistocene EAE are elevated relative to almost all of the recent human samples. The frequencies for the presence of any grade of EAE, including Xuchang 1 and 2 and Xujiayao 15 in the archaic sample, are 61.1% (n = 18) for the archaic sample and 58.3% (n = 12) for the early modern humans (Table 2). These values are exceeded by only two recent human samples (Fig 9), one from coastal Brazil and one from the Canary Islands [43,44]. If two of the eastern Eurasian archaic crania with very small EAE are considered to lack them (Ngandong 6 and ZKD Skull 12), and if the same approach is applied to the ZKD-UC 102 and Jingcuan 1 early modern human crania, the eastern Pleistocene frequencies decrease to 50.0% and 41.7%. These values still place them above all of the recent human interquartile ranges, within the ranges of the low and middle latitude “wet” samples, but above the other sample distributions (Fig 9). These more conservative lower frequencies are matched or approached (>40% EAE) by only 6 (5.3%) of the recent humans samples.

## Discussion

The presence of these exostoses in the Xuchang and Xujiayao auditory canals, the high frequencies of them in the eastern Eurasian later Pleistocene samples, and the sizes and forms of the ones in Xuchang 1 and 2 all have broader implications. In particular, they relate to levels of aquatic proximity and resource exploitation and reduction in auditory acuity.

### EAE and cold water

As summarized by Villotte and Knüsel [6], there is abundant evidence that the osseus development of EAE is greatly enhanced by exposure to cold water. In particular, frequent exposure of the canal to cold water leads to subperiosteal vasodilation, increased tension in the periosteum, and consequent bone deposition ([45]; see [6,8]). As a result, extant humans who engage in a variety of cold water activities, including swimming, surfing, kayaking and diving, exhibit a marked elevation in the formation of EAE (hence “swimmer’s ear,” “surfer’s ear” and “diver’s ear”) (e.g., [6,40,46–50]). In addition, among recent human archeological samples with evidence of aquatic resource exploitation, there is often an elevated frequency of EAE (e.g., [42,43,51–54]). The cold water exposure can consist of immersion of the head during activities and/or damp and cold wind [45–50], and therefore the presence of EAE may reflect aquatic activities and/or proximity to wetlands. Although the precise pathophysiology of their formation is poorly known [8], some form of auditory canal irritation (of which cold water is by far the best documented) appears to be involved. There have been suggestions of an inherited component being involved (at least in terms of susceptibility) [3,55], but its effects on population frequencies are likely to be minor (see discussions in [4–6]).

This global pattern was summarized by Kennedy [4], in which she found the highest frequency of EAE among middle latitude (30°–45°) samples, arguing that higher latitude populations would avoid exposure to the very cold water and lower latitude water would be insufficiently cold to induce EAE. Further analyses and additional data (S2 Text), with latitudinal samples separated into “wet” and “dry” contingents (see above and S2 Text), provides a slightly modified pattern (Fig 9). High latitude EAE remain infrequent, as do low latitude “dry” ones. Yet, a number of “wet” samples from both low and middle latitudes provide higher frequencies. Their frequency distributions are not significantly different from the middle latitude “dry” one, but they are distinctly separate from the low latitude “dry” samples, and the middle latitude “wet” distribution is substantially different from both high latitude ones (Table 3). This global pattern therefore further supports the clinical and paleopathological observations in indicating a predominant role for aquatic/wetland exposure in EAE formation, but especially in the lower latitudes where the “wet” and “dry” frequency distributions are significantly different.

**Table 3:** Kruskal-Wallis Z-values for two-tailed pairwise comparisons across the six sets of recent human external auditory exostosis (EAE) frequencies. With a Bonferroni correction, the medians are significantly different if Z > 2.935. Overall Kruskal-Wallis p < 0.0001 (corrected for ties; χ^2^ = 37.59). Normality (both skewness and kurtosis) rejected at p < 0.0001.

	Low “wet”	Low “dry”	Middle “wet”	Middle “dry”	High “wet”
Low “dry”	3.345				
Middle “wet”	2.594	5.203			
Middle “dry”	0.242	3.225	2.183		
High “wet”	2.555	0.259	4.383	2.554	
High “dry”	1.831	1.133	3.844	1.880	0.754

It is therefore of interest that the eastern Eurasian later Pleistocene humans exhibit an elevated frequency of EAE and of the higher grades of EAE (Table 2; Figs 8 and 9). Among the early modern humans, the only maritime sample is the Minatogawa one, and all three of the individuals preserving auditory canals exhibit EAE (S1 Text). EAE are, however, also present on four of the other early modern human crania, none of which derive from coastal sites but one derives from a river terrace (Table 1). Moreover, there is early Upper Paleolithic evidence for coastal adaptations from Okinawa and further south [56–59], as well as isotopic evidence of aquatic resource utilization inland in northern China [60]. Among the archaic humans, the Xuchang, Xujiayao, Dali, Yunxian and Ngandong fossils were directly associated with water lain sediments (Table 1); the Xuchang site formed in spring lacustrine deposits [19], and the Xujiayao, Dali, Yunxian and Ngandong sediments are fluviatile or fluviolacustrine [25,61–63]. Archeological evidence for aquatic resource exploitation at Xuchang, Xujiayao and the eastern Middle Pleistocene sites is unavailable, but inland and coastal aquatic resources were exploited in the late Middle and earlier Late Pleistocene of western Eurasia and Africa [64–70], making it likely that they were utilized as well in the east. It therefore appears reasonable to infer that the elevated frequencies of EAE in the eastern Eurasian samples are due, at least in part, to maritime or riverine/lacustrine proximity and activities, most likely related to the exploitation of aquatic resources.

It should nonetheless be noted that such an inference is based on the overall pattern of these geographically and temporally pooled samples. As emphasized by Villotte and Knüsel [6], “EAE should not be used as a marker of aquatic activities in case studies of single prehistoric individuals.” In addition, all of the Chinese archaic humans are from middle latitude sites (albeit some close to 30°; Hexian and Yunxian: ≈31°, Xuchang: ≈34°, Dali: ≈35°), for which the recent human “wet”/”dry” EAE frequency distributions extensively overlap (Fig 9) and are not significantly different (Table 3). And the evidence for infection on the superior auditory porus of Xujiayao 15, in the form of the porous new bone (Fig 7C), may indicate a non-aquatic origin of its auditory canal changes. Yet, the overall pattern is sufficiently pronounced, for both the eastern Eurasian archaic and early modern human EAE, to argue for a substantial contribution of exposure to cold water.

### EAE and auditory acuity

The degree of development of some of the eastern Eurasian Pleistocene EAE, and in particular those of Xuchang 1 and 2, also has implications for their hearing abilities. The more advanced stages of EAE are associated with conductive hearing loss (CHL) in extant humans [2,40,47,48,71–74]. Most EAE are located laterally in the auditory canal, and therefore they do not usually impinge directly on the tympanic membrane. Yet, they may extend medially, causing stenosis of the canal (atresia) and associated CHL [75]. Moreover, large (Grade 3) EAE make it extremely difficult for the normal irrigation of the ear canal to cleanse the cerumen and exogenous debris from the canal [8,74,76]. The accumulated material then reduces both the sound transmission through the ear canal and the ability of the tympanic membrane to transmit the sound waves to the middle ear [77,78]. Accumulated cerumen and exogenous material in the canal is therefore a common cause of EAE related CHL in recent humans.

The Grade 2 EAE of Xuchang 1 are likely to have caused only minor CHL, since they produced only minor atresia of the canal and (unless there were large ones on the missing tympanic bones) are not likely to have greatly inhibited normal meatal irrigation. The larger left and especially right Grade 3 ones of Xuchang 2, with the apparent stenosis, would have impeded such cleansing of the canal. In addition, all of the Xuchang EAE extended to the attachment area for the tympanic membrane. To the extent that they impinged on that membrane (most evident on the right ones), they would have reduced the ability of sound waves to be transmitted to the ossicles of the middle ear and further promoted CHL.

Consequently, Xuchang 2 appears to minimally have had an advanced degree of unilateral conductive hearing loss and probably some CHL on both sides. Xuchang 1 probably also had some degree of CHL. In addition to a general reduction in hearing acuity, unilateral CHL limits one’s ability to discern the signal from background noise and to locate sounds in space. Among modern urban children it affects cognitive development [79]; it is likely to have been a much more serious impairment among Pleistocene foragers given its relationship to hunting effectiveness in recent human foragers [80]. Effective hearing is also an important component in learning lithic (and other) technology, given the need for auditory feedback [81]. In addition to a reduced effectiveness in communication and coordinated social activities, it would have made the individuals vulnerable to the ubiquitous medium to large carnivores of Late Pleistocene Eurasia [82], including early Late Pleistocene China; *Pachycrocuta* cf. *sinensis*, *Ursus* sp. and *Viverra* cf. *zibetha* are represented at Xuchang (including coprolites of the first and canine marks on faunal remains), and *Canis lupus* and *Panthera* cf. *tigris* were present at Xujiayao [21,22,83,84]. In addition, both of these sites preserve abundant remains of a suite of large and potentially dangerous herbivores, including *Palaeoloxodon* sp., *Coelodonta antiquitatis*, *Dicerorhinus mercki*, *Equus ferus*, *E*. *hemionus*, *Bos primigenius* and *Megaloceros ordosianus*.

The pronounced EAE of Xuchang 2 in particular (and to a lesser extent that of Xuchang 1) therefore indicate severely compromised auditory abilities. These impairments would have impeded the individuals’ abilities to participate in socially coordinated activities and would have made them vulnerable to large mammals, both carnivorous and herbivorous. The EAE therefore imply some level of social support to enable their survival in their Pleistocene contexts. As such, Xuchang 1 and 2 join a series of other Pleistocene individuals with serious developmental or degenerative abnormalities (see list in [85]), including two others with CHL (Atapuerca-SH Cr.4 and Shanidar 1) [12,14]. Most of these abnormalities would have impeded their abilities to function fully in a Pleistocene foraging context (e.g., [14,86–89]), and many would have required some level of social support beyond the mother-child dyad for long term survival (see the extended discussion in [14]).

## Conclusions

The early Late Pleistocene Xuchang 1 and 2 and Xujiayao 15 late archaic humans provide evidence of external auditory exostoses (EAE), minor in the last, moderate in the first, and pronounced in Xuchang 2. As such, they join a suite of eastern Eurasian Pleistocene archaic and early modern humans with minor to moderate EAE, both samples presenting overall frequencies at the top of recent human distributions. The Xujiayao 15 one is associated with auditory porus new porous bone which may indicate an infectious origin for the EAE, but the other fossils are likely to have developed EAE from generalized inflammation of the meatus. The most likely stimulant, based on recent clinical and bioarcheological data, is cold water from aquatic proximity and/or activity, presumably for resource exploitation among these Pleistocene humans. In addition, the pronounced EAE of Xuchang 2 in particular implies conductive hearing loss and consequent social support for coordinated activities and protection from the dangers of a Pleistocene foraging existence.

## Supporting information

S1 Text External auditory exostoses in eastern Eurasian humans. (PDF)

S2 Text External auditory exostoses in recent human samples. (PDF)
