# Supplementary material for: External auditory exostoses in the Xuchang and Xujiayao human remains: Patterns and implications among eastern Eurasian Middle and Late Pleistocene crania
Source: PLoS One. 2017 Dec 12;12(12):e0189390. doi: 10.1371/journal.pone.0189390 (PMC5726651; doi:10.1371/journal.pone.0189390)
Supplement: S1 Text — (PDF) [file pone.0189390.s001.pdf]

External Auditory Exostoses in the Xuchang and Xujiayao Human Remains: Patterns and Implications among Eastern Eurasian Middle and Late Pleistocene Crania
Supporting Information

Erik Trinkaus[1] and Xiujie Wu[2]

[1] Department of Anthropology, Washington University, Saint Louis MO 63130, USA; trinkaus@wustl.edu
[2] Key Laboratory of Vertebrate Evolution and Human Origins, Institute of Vertebrate Paleontology and Paleoanthropology, Chinese Academy of Sciences, Beijing 100044, China. wuxiujie@ivpp.ac.cn

S1 Text: External auditory exostoses in eastern Eurasian humans

Tables S1 and S2 provide external auditory exostosis (EAE) observations on Middle Pleistocene archaic human crania and Late Pleistocene early modern human crania. Unless otherwise referenced, the observations were made by the authors on the original fossil specimens.

S1 References

1. Weidenreich F. Morphology of Solo man. Anthropol Pap Am Mus Nat Hist. 1951; 43(3):222-90.
2. Santa Luca AP. The Ngandong fossil hominids. Yale Univ Pub Anthropol. 1980; 78:1-175.
3. Weidenreich F. The skull of *Sinanthropus pekinensis*. Palaeontol Sinica. 1943; 10D:1-485.
4. Suzuki H. Skulls of the Minatogawa man. Bull Univ Mus Univ Tokyo. 1982; 19:7-49.

~~Table S1~~. External auditory exostoses in eastern Eurasian Middle Pleistocene archaic humans.

	Side	***EAM exostoses***	***Grade***	***Source***[1]
Dali 1	rt	very small protrusion extending in from anterosuperior meatus, and small rounded knob in from posteroinferior tympanic	1	CT
	lt	distinct rounded growth in from posterosuperior meatus inside of porous	1	CT
Hexian 1	rt	absent	0	
	lt	absent	0	
Jinniushan 1	rt	absent	0	cast
Ngandong 1	rt	absent	0	[1,2]
Ngandong 6	lt	posteroinferior inward swelling	1	[1,2]; cast
Ngandong 7	rt	absent	0	[1,2]
	lt	absent	0	[1,2]
Ngandong 10	rt	anteroinferior protrusion	1	[1,2]
Ngandong 11	rt	absent	0	[1,2],cast
	lt	absent	0	[1,2],cast
Ngandong 12	rt	absent	0	[1,2]
	lt	posterior tympanic small exostosis	1	[1,2]
Yunxian	lt	small rounded knob in anterosuperior meatus, and several small rounded knobs in posterior superior and inferior meatus.	1	
ZKD Skull 5	rt	small knob anterosuperior and anteroinferior thickening with modest protrusion into meatus	1	[3]; cast
	lt	absent	0	[3]
ZKD Skull 10	rt	distinct superoanterior knob from anterior tympanic, into canal	2	[3]; cast
ZKD Skull 11	lt	absent; inferior tympanic thickening	0	[3]; cast
ZKD Skull 12	lt	anteroinferior thickening with a broad bulge into the meatus	1	[3]; cast

[1] Unless otherwise indicated, observations are on the original specimens. CT: from CT scan of cranium; cast: from a cast of the specimen, taking into account any casting artifacts;

Table S2. External auditory exostoses in eastern Eurasian Late Pleistocene early modern humans.

	Side	***EAM Exostoses***	***Grade***	***Suorce***[1]
Gezi 1	rt	absent	0	
Jingcuan 1	rt	very small swelling in mid-posterior meatus	1	
Lijiang	rt	absent	0	
Liujiang 1	rt	small swelling in posterior mid-superior meatus	1	CT
	lt	distinct small rounded protrusion from posterior meatus	1	CT
Minatogawa 1	rt	posteroinferior moderate cylinder along meatus	1	[4]; cast
	lt	absent	0	[4]; cast
Minatogawa 2	rt	medium growths mid-posterior and posteroinferior	2	[4]
Minatogawa 4	rt	three small knobs posterosuperior, posteroinferior and anteroinferior	1	[4]
Wajak 1	rt	absent	0	
ZKD-UC 101	rt	absent	0	cast
	lt	absent	0	cast
ZKD-UC 102	rt	small swelling on anterosuperior tympanic edge	1	cast
ZKD-UC 103	rt	small knobs posteroinferior and anterosuperior	1	cast
	lt	absent	0	cast
Ziyang 1	lt	absent	0	

[1] Unless otherwise indicated, observations are on the original specimens. CT: from CT scan of cranium; cast: from a cast of the specimen, taking into account any casting artifacts;
