# Supplementary material for: External auditory exostoses in the Xuchang and Xujiayao human remains: Patterns and implications among eastern Eurasian Middle and Late Pleistocene crania
Source: PLoS One. 2017 Dec 12;12(12):e0189390. doi: 10.1371/journal.pone.0189390 (PMC5726651; doi:10.1371/journal.pone.0189390)
Supplement: S2 Text — (PDF) [file pone.0189390.s002.pdf]

External Auditory Exostoses in the Xuchang and Xujiayao Human Remains: Patterns and Implications among Eastern Eurasian Middle and Late Pleistocene Crania
Supporting Information

Erik Trinkaus[1] and Xiujie Wu[2]

[1] Department of Anthropology, Washington University, Saint Louis MO 63130, USA; trinkaus@wustl.edu
[2] Key Laboratory of Vertebrate Evolution and Human Origins, Institute of Vertebrate Paleontology and Paleoanthropology, Chinese Academy of Sciences, Beijing 100044, China. wuxiujie@ivpp.ac.cn

S2 Text: External auditory exostoses in recent human samples

~~Table S3~~. Distribution of external auditory exostosis grades among recent human samples providing grades of severity.

	Grade 0	*Grade 1*	*Grade 2*	*Grade 3*	*N*	*Ref*
Vaud/Valais (pooled) ("dry")[1,2]	100%	--	--	--	83	[1]
Khoisan ("dry")	100%	--	--	--	123	[2]
Gurgy ("dry")[2]	97.1%	2.9%	--	--	35	[1]
Stuttgart-Mülhausen ("dry")[2]	95.6%	4.4%	--	--	45	[1]
New Guinea coast (south) ("wet")	96.7%	--	3.3%	--	92	[2]
Melanesian (north) ("wet")	95.3%	4.7%	--	--	43	[2]
Santa Rosa Island (pooled) ("wet")	89.8%	7.2%	2.4%	0.6%	166	[3]
Chile-Late Period ("wet")	78.2%	11.9%	5.9%	4.0%	101	[4]
Muge (pooled) ("wet")[2]	76.0%	22.0%	2.0%	--	50	[1]
Iron Gates (pooled) ("wet")[2]	75.2%	20.8%	4.0%	--	101	[1]
Chile-Archaic Period ("wet")	72.3%	22.3%	4.3%	1.1%	94	[4]
Isola Sacra ("wet")	68.8%	20.8%	6.3%	4.2%	48	[5]
Chile-Formative ("wet")	61.3%	21.3%	12.0%	5.3%	75	[4]
"Dry" average	98.2%	1.8%	0.0%	0.0%		
"Wet" Average	79.3%	14.6%	4.5%	1.7%		

[1] See Table S5 for explanation and justification of "wet'/"dry" attributions.
[2] Data from the side with the largest sample.

~~**Table S4**~~. Frequencies of external auditory exostosis (EAE) presence in samples of recent humans. The samples are grouped into low (<30°), middle (30° - 45°) and high (5°) latitude samples following Kennedy [6], and within each, into “wet” (coastal and riverine) and “dry” (inland with little or no aquatic exploitation). Data from Kennedy [6] supplemented with additional data.

The samples only include adults and later adolescents (>≈15 years). Male and female samples are combined, because sex is not known for most of the Pleistocene specimens. The data are also not separated by sex for a substantial number of the recent human samples. It is nonetheless recognized that a number of studies (e.g., [1,3,4,7-9]) have found substantial differences in the male versus female frequencies, with the males often having higher the higher incidence. Small (N < 30) samples are not included.

In the allocation of the samples into the “wet” versus “dry” categories, several factors were employed. In cases in which the authors have provided explicit contrasts between coastal/riverine versus terrestrial residence/occupation/resource exploitation (e.g., [1,3,4,8,10,11]), their divisions have been followed. For inland localities with little or no evidence of aquatic resource exploitation, the samples have been considered “dry.” Yet, inland (riverine) sites are considered “wet” where there is evidence of aquatic resource exploitation (e.g., Iron Gates Mesolithic sites, Indian Knoll). Coastal samples, even if there is no associated evidence for the exploitation of littoral resources, have been placed in the “wet” category, given the effects of general maritime exposure and associated wind chill [8,12]. Pooled samples based on national boundaries, immigrant groups, and pooled samples from very large islands with both coastal and inland areas are not included. It is fully recognized that some of the samples considered to be “wet” or “dry” could be placed in the other category. However, such resorting is not likely to substantially alter the “wet”/”dry” distributions within each of the latitudinal zones.

It is also recognized that the laritudinal zones, with cut-offs at 30° and 45°, are partially arbitrary, especially given variation in sea temperatures at a given latitude due to major oceanic currents. For those samples which fall close to a latitudinal boundary (e.g., Chinook, Arikara), they have been placed in the higher latitude sample. These comments only serve to reinforce that such a global analysis, in contrast to detailed ones within regions, primarily serve to highlight overall patterns.

Site	*Frequency*	*N*	*Reference*
**Low Latitude (<30°) “dry”**			N = 22
Australia-North	1.5	172	[6]
Australia-Central	3.2	127	[6]
Australia-Queensland	4.5	110	[6]
Australia-North Territory	0.7	132	[6]
Australia-North Queensland	0.0	54	[6]
Punjab	0.0	53	[6]
Lachish, Israel	0.0	695	[6]
Egypt XX Dynasty	1.8	379	[6]
Egypt XXI Dynasty	2.7	75	[6]
Egypt Pre-dynastic	0.0	60	[6]
Egypt Middle Kingdom	0.0	182	[6]
Egypt Late period Giza	0.0	50	[6]
Canary Islands Interior	0.0	45	[11]
Canary Islands Highlands	0.9	226	[13]
Nubia-Jebel Moya	0.0	32	[6]
Nubia-Historic	1.2	431	[14]
Nubia-Kerma	0.4	224	[14]
Ashanti	0.0	56	[6]
Khoisan	0.0	123	[2]
Ayalan, Equador	2.9	103	[60]

Botocudo, Brazil	2.5	40	[8]
Cerca Grande, Brazil	2.0	50	[8]
**Low Latitude (<30°) "wet"**			N = 30
Hawai-Oahu	0.0	1063	[6]
Hawaii-Mokapu	13.2	49	[6]
Hawaii-pooled	20.3	148	[6]
New Britain	0.0	85	[6]
New Ireland	0.0	53	[6]
Solomon Islands	0.0	50	[6]
New Caledonia	2.9	85	[6]
New Hebrides	0.0	84	[6]
Fiji	0.0	32	[6]
Society Islands	0.0	58	[6]
Lesser Sundas	0.0	45	[6]
Easter Island	8.6	64	[6]
Marquesas	2.8	51	[6]
Marquesas	18.2	36	[6]
Southern Cook Islands	3.8	52	[15]
Samoa	25	38	[15]
Island Melanesia	3.1	32	[15]
Duff Islands	6.1	59	[15]
New Guinea South Coast	3.3	95	[2]
New Guinea North/Melanesia	3.6	44	[2]
Canary Islands	64.7	34	[16]
Canary Islands Coast	40.2	97	[13]
Canary Islands fishing	8.6	105	[11]
Corondó, Brazil	0.0	32	[8]
Guaraguaçu, Brazil	13.3	30	[8]
Moro do Ouro, Brazil	18.9	37	[8]
Rio Comprido, Brazil	54.8	31	[8]
Base Aérea, Brazil	66.8	36	[8]
Tapera, Brazil	28.6	70	[8]
Cabeçuda, Brazil	43.2	74	[8]
**Middle Latitude (30° - 45°) "dry"**			N = 19
Australia-Murray Valley	27.9	476	[6]
Australia-Murray Valley	21.2	99	[6]
Tasmania	4.8	62	[6]
Tasmania	9.0	67	[6]
Jomon, Japan	18.7	542	[15]
Yayoi, Japan	18.9	90	[15]
Çayönü, Turkey	17.5	97	[17]
Hopewell Mounds, IL	34.1	41	[6]
Klunk II, IL	34.0	78	[6]
Woodland, IL	2.0	50	[6]
Texas pooled	10.3	348	[18]
Pecos Pueblo, NM	2.4	500	[6]
Gran Quivira, NM	3.0	35	[6]
Grasshopper, AZ	0.0	161	[6]
Point of Pines, AZ	4.9	82	[6]
Turkey Creek, AZ	0.0	104	[6]
Pyramid Lake, NV	0.3	59	[6]
North Chile highland	0.0	549	[4]
North Chile valley	2.3	264	[4]

**Middle Latitude (30° - 45°) "wet"**			N = 14
Iron Gates pooled	29.4	126	[1]
Indian Knoll, KY	49.6	474	[6]
Isola Sacra	12.5	957	[9]
Isola Sacra	31.3	48	[5]
Velia	18.6	348	[9]
Muge pooled	19.4	72	[1]
Sado pooled	9.1	33	[1]
Vlasac Iron Gates	34.2	38	[7]
Santa Rosa Island early	8.1	62	[15]
Santa Rosa Island middle	13.9	72	[15]
Santa Rosa Island late	9.6	73	[15]
North Chile fertile coast	30.6	284	[4]
North Chile dry coast	30.8	52	[4]
Argentine wetlands	6.3	176	[19]
**High Latitude (>45°) "dry"**			N = 16
North China	0.0	100	[6]
Ainu Hokkaido	1.6	128	[6]
Stuttgart-Mülhausen	5.0	60	[1]
Gurgy, France	2.1	48	[1]
Vaud-Valais	0.0	94	[1]
Southeast Scotland	0.0	50	[6]
York, UK	0.0	52	[6]
Hythe, UK	0.0	50	[6]
Salish interior	3.4	87	[6]
Inuit Yukon	2.0	50	[6]
Inuit St. Lawrence	2.0	50	[6]
Arikara/Mandan	21.1	109	[20]
Arikara-Crow Creek	2.3	613	[6]
Arikara-Mandan	8.8	34	[6]
Mandan	4.4	45	[6]
Tierra-del-Fuego inland	1.9	53	[10]
**High Latitude (>45°) "wet"**			N = 13
Hebrides	0.0	50	[6]
Shetlands	0.0	50	[6]
Iceland	0.0	82	[6]
Greenland	0.0	51	[6]
Inuit coastal	0.0	50	[6]
Pre-Aleut, AK	0.0	47	[6]
Aleut, AK	0.0	50	[6]
Salish coastal	6.5	107	[6]
Koskimo, BC	0.8	143	[6]
Cowichan, BC	2.1	117	[6]
Haida, BC	0.0	36	[6]
Chinook, WA/OR	27.7	83	[6]
Tierra-del-Fuego coastal	9.1	55	[10]

S2 References

1. Villotte S, Stefanović S, Knüsel CJ. External auditory exostoses and aquatic activities during the Mesolithic and the Neolithic in Europe: results from a large prehistoric sample. Anthropol (Brno). 2014; 52(1):73-89.
2. Ponzetta MT, Hauser G, Vienna A. Auditory hyperostosis and the environment: an update. Int J Anthropol. 1997; 12(2): 29-42.
3. Kuzminsky SC, Erlandson JM, Xifara T. External auditory exostoses and its relationship to prehistoric abalone harvesting on Santa Rosa Island, California. Intl J Osteoarchaeol. 2016; 26:1014-23. doi: 10.1002/oa.2512.
4. Standen VG, Arriaza B. Santoro CM. External auditory exostosis in prehistoric Chilean populations: a test of the cold water hypothesis. Am J Phys Anthropol. 1997; 103(1):119-29.
5. Manzi G, Sperduti A, Passarello P. Behavior-induced auditory exostoses in Imperial Roman society: Evidence from coeval urban and rural communities near Rome. Am J Phys Anthropol. 1991; 85:253-260.
6. Kennedy GE. The relationship between auditory exostoses and cold water: a latitudinal analysis. Am J Phys Anthropol. 1986; 71(4):401-15.
7. Frayer DW. Auditory exostoses and evidence for fishing at Vlasac. Curr Anthropol. 1988; 29(2):346-349.
8. Okamura MMM, Boyadjian CHC, Eggers S. Auditory exostoses as an aquatic activity marker: A comparison of coastal and inland skeletal remains from tropical and subtropical regions of Brazil. Am J Phys Anthropol. 2007; 132: 558-67. doi:10.1002/ajpa.20544.
9. Crowe F, Sperduti A, O'Connell TC, Craig OE, Kirsanow K, Germoni P, et al. Water-related occupations and diet in two Roman coastal communities (Italy, first to third century AD): correlation between stable carbon and nitrogen isotope values and auricular exostosis prevalence. Am J Phys Anthropol. 2010; 142(3):355-66. doi: 10.1002/ajpa.21229.
10. Ponce P, Ghidini G, González-José R. External auditory exostosis “at the end of the world”: the southernmost evidence according to the latitudinal hypothesis. Brit Archaeol Rep. 2008; S1743: 101–7.
11. Arnay-de-la-Rosa M, González-Reimers E, Velasco-Vázquez J, Santolaria-Fernández F. Auricular exostoses among the prehistoric population of different islands of the Canary archipelago. Ann Otol Rhinol Laryngol. 2001; 110(11):1080-3.
12. Hurst W, Bailey M, Hurst B. Prevalence of external auditory canal exostoses in Australian surfboard riders. J Laryngol Otol 2004; 118:348-31.
13. Velasco-Vázquez J, Betancor-Rodriguez A, Arnay-de-la-Rosa M, Gonzalez-Reimers E. Auricular exostoses in the prehistoric populaion of Gran Canaria. Am J Phys Anthropol. 2000; 112:49-55.
14. Godde K. An examination of proposed causes of auditory exostoses. Int J Osteoarchaeol. 2010; 20:486-490. doi: 10.1002/oa.1058.
15. Katayama K. Auditory exostoses among ancient human populations in the Circum-Pacific area: Regional variation in the occurrence and its implications. Anthropol Sci. 1998; 106(4): 285-96.
16. Dutour O, Onrubia-Pintado J. Interactions homme-environnement océanique pendant la préhistoire récente des Iles Canaries: nouvelles données paléoanthropologiques de la région de Galdar (Grande Canarie). C R Acad Sci Paris Série III, 1991; 313:125-30.
17. Özbek M. Auditory exostoses among the prepottery Neolithic inhabitants of Çayönü and Aşikl, Anatolia; its relation to aquatic activities. Int J Paleopathol. 2012; 2:181-186. doi: org/10.1016/j.ijpp.2012.10.004.
18. Goldstein MS. Skeletal pathology of early Indians in Texas. Am J Phys Anthropol. 1957; 15(3):299-311.

19. Mazza B. Auditory exostoses in Pre-Hispanic populations of the Lower Paraná wetlands, Argentina. Int J Osteoarchaeol. 2016; 26:420-430. doi: 10.1002/oa.2432.
20. Gregg JB, McGrew RN. Hrdlička revisited (external auditory canal exostoses). Am J Phys Anthropol. 1970; 33:37-40.
